# 

**Published:** 1860-05

**Authors:** 


### RECEIPTS FOR THE JOURNAL, FOR APRIL 1860.

| | | |
|---|---|---|
| Benj. Keith, Ind | Vol. 3 | 2 00 |
| J. N. Allen, Ill. | " 2 | 2 00 |
| John F. Summers, Ind | " 3 | 2 00 |
| B. Wilson, Ill | " 3. | 2 00 |
| Blexton Harris, Ill | " 3 | 2 00 |
| J. J. M. Angean, Wis | " 3 | 2 00 |
| Joseph Slack, Ill | " ¼ of 3 | 50 |
| W. R. Griswold, Ill | " ½ of 3 | 1 00 |
| Hays McKinley, Wis | " 3 | 2 00 |
| Amos Crosby, Mich | " 3 | 2 00 |
| J. B. Moffett, Wis | " 3 | 1 00 |
| A. M. Golliday, Iowa | " 3 | 2 00 |
| R. E. McVey, Ill | Vol. 3. | 2 00 |
| John W. Xeunce, Ill | " ½ of 3 | 1 00 |
| John E. Ennis, Iowa | " 3 | 2 00 |
| Frederick Bartells, Ill | " 3 | 2 00 |
| James Carson Iowa | " 2½ to 3½ | 2 00 |
| James Johnson, Ind | " 3 | 2 00 |
| Samuel A. Covert, Ind | " 2 | 2 00 |
| C. Nettleton, Wis | " 3 | 2 00 |
| A. Wood. Ind | " 2 | 1 00 |
| Wm. Watsan, Iowa | " 3 | 2 00 |
| C. C. Allen, Ill | " 3 | 2 00 |

☞ The Iowa State Medical Society will hold its next Annual Meeting in the City of Dubuque, on the second Wednesday of May next. All Physicians and Surgeons in good standing, residing within the State, who are authorized to practice Medicine and Surgery by any Medical College qualified to grant Diplomas, and acknowledged by the American Medical Association as such, are eligible to be elected as Members of the State Society.

All members of the profession in the State who come within the above requirements, are invited to meet and become connected with the Association in conformity with its code of Regulations.

A. PHILLIPS,

*Sec'y Iowa State Med. Society.*

*Dubuque, March*, 30, 1860.

